# Thermal Performance of Wood Frame Construction with Phase Change Material in the Brazilian Subtropical Climate

**DOI:** 10.3390/ma18030681

**Published:** 2025-02-04

**Authors:** Julia Vieira, Raquel Oliveira, Ana Abreu, Marcin Różycki, Tomasz Niemiec, Mateusz Sitarz

**Affiliations:** 1 Post-Graduate Program in Civil Engineering, Federal Center for Technological Education of Minas Gerais, Belo Horizonte 30510-000, Brazil; raqueldiniz@cefetmg.br; 2 Department of Structures, Institute of Structures and Transport, Faculty of Engineering, Universidad de la República, Montevideo 11300, Uruguay; aabreu@fing.edu.uy; 3 Faculty of Civil Engineering, CUT Doctoral School, Cracow University of Technology, 31-155 Cracow, Poland; marcin.rozycki@doktorant.pk.edu.pl (M.R.); tomasz.niemiec@doktorant.pk.edu.pl (T.N.); 4 Chair of Building Materials Engineering, Faculty of Civil Engineering, Cracow University of Technology, 31-155 Cracow, Poland; mateusz.sitarz@pk.edu.pl

**Keywords:** thermal storage materials, lightweight construction, thermal insulation, thermal comfort, unconditioned building, temperate oceanic climate

## Abstract

In a Brazilian subtropical climate, Wood Frame construction, valued for sustainability and thermal inertia, is being tested for compatibility with Phase Change Materials (PCMs) to improve thermal performance. This study addresses the lack of research on these technologies in Brazil and evaluates the thermal performance of a single-story Wood Frame housing envelope with and without PCM in Curitiba-PR, located in southern Brazil with Cfb climate classification. Dynamic energy simulation followed ASHRAE Standard 55-2017 criteria for occupant thermal comfort. The results indicated that integrating PCM with thermal insulation (EPS) significantly improved thermal performance, reducing the daily indoor temperature range by up to 6.4 °C and increasing comfortable hours by 20%. However, Wood Frame construction without either BioPCM or EPS proved inadequate in achieving the minimum level of thermal performance required by Brazilian standards. This underscores the importance of evaluating potential users’ thermal comfort conditions alongside the building’s overall thermal performance. It also emphasizes the need to carefully consider the level of thermal insulation in conjunction with PCM for effective design decisions. Thus, this study promotes the integration of PCM and thermal insulation to enhance thermal comfort and sustainability in Wood Frame constructions in the subtropical climate of Brazil.

## 1. Introduction

In the coming decades, a significant increase in cooling demand for residential areas is anticipated, especially in developing regions with warm climates, owing to global warming [1]. Conversely, even in the coldest regions of Brazil, the demand for heating is predicted to nearly cease by 2080 [2]. Demographic factors and the growing need for occupants’ thermal comfort in buildings are expected to trigger a significant rise in the energy demand for these systems by 2050 [3].

To address these challenges, strategies like thermal energy storage (TES) are being implemented to enhance the thermal performance of buildings, aligning with local climate conditions and promoting energy efficiency [4]. Within the realm of TES technology, Phase Change Materials (PCMs) are currently the subject of intensive research due to their capability to store thermal energy in a latent state [5].

PCMs can absorb heat during the transition from a solid to a liquid state as temperatures rise, and release heat during solidification as temperatures drop [6]. Integrating PCMs into buildings allows for the direct storage of solar heat within walls, ceilings, and floors, facilitating the passive regulation of indoor temperatures during phase changes [7]. When properly implemented, PCMs minimize indoor temperature fluctuations, sustaining temperatures close to the desired level over extended periods, thereby enhancing occupants’ thermal comfort conditions and improving building energy efficiency [8].

Various techniques exist for incorporating PCM into building components, including plaster, wood, brick, concrete, and vermiculite. Furthermore, prefabricated commercial products, like gypsum boards and PCM-filled containers, are readily available for direct installation in walls, roofs, ceilings, floors, glass windows, and other building elements [9]. Moreover, they can be integrated into thermal insulation materials within building envelopes to enhance thermal insulation performance and improve energy efficiency [10].

Nonetheless, the efficacy of PCMs in buildings hinges on several factors, including volume, thermophysical properties, climate, architectural design, construction materials, occupancy patterns, and the operation of the HVAC system [11]. Local climatic conditions, categorized based on temperature and precipitation ranges, significantly influence this efficacy, and are necessary for considering factors such as latitude, altitude, solar radiation, humidity, wind characteristics, and amount of cloud cover [12,13,14]. The optimization of the storage effect entails adjusting the type, quantity, and methods of PCM incorporation in construction [15], with temperature ranges and enthalpy of fusion being key determinants [16]. Ensuring that the melting/freezing temperature of the PCM closely aligns with the desired room temperature is recommended for the precise control of internal temperature within the range of user-acceptable thermal comfort [17,18]. Moreover, diurnal temperature fluctuations should allow for phase change cycles [15]. Some studies suggest that the phase change temperature of PCMs should be slightly higher than the average indoor temperature—typically by 1 to 3 °C in environments without PCMs—to optimize daytime heat storage [19]. Conversely, other research recommends that PCM melting temperatures should be 3 to 5 °C higher than the average outdoor air temperature to maximize indoor thermal comfort [20]. However, excessively high melting temperatures may lead to elevated nighttime indoor temperatures, increasing thermal discomfort compared to buildings without PCMs. A comprehensive analysis of these factors is essential for the proper selection of PCMs to meet application requirements [10]. Numerical modeling serves as an important tool in providing accurate evidence of the potential economic and environmental benefits of these systems [21,22].

In the context of warm temperate climates, PCM incorporation into building envelopes holds significant promise for enhancing thermal comfort conditions and achieving energy savings indoors [10]. For instance, in an Australian city (Cfb climate classification), integrating PCMs with varying melting temperatures into the internal surface of residential building roofs reduced indoor temperature fluctuations by 1.5 to 3.5 °C [12]. Similarly, optimized PCM integration in unconditioned residential building envelopes in the same climate reduced the risk of severe heat stress by up to 65% [23]. In mid-rise apartment buildings in Cfb regions of Australia, Spain, and New Zealand, annual energy savings of up to 13.6% were achieved, with a payback period ranging from 7 to 24 years and maximum annual carbon emission reduction of 6987 kg CO_2_-eq/kWh [24].

Moreover, natural ventilation techniques have shown significant potential in enhancing PCM cooling energy consumption in climates with low outdoor temperatures, such as Curitiba (Cfb) in southern Brazil. These techniques reported energy savings of 37.3% when PCM was combined with night ventilation and 65.7% when coupled with temperature-controlled ventilation, with a payback period of up to 9 years and a potential CO_2_ emission reduction of up to 274.5 tons over 50 years [25].

However, the efficacy of PCMs varied across different scenarios. In unconditioned buildings in Poland, PCM effectiveness fluctuated during the summer, and a single optimal melting temperature range was insufficient [26]. Similarly, PCMs showed limited energy-saving efficiency during Australian winters [12]. In a UK house (Cfb), incorporating PCMs into external heavyweight walls reduced the occurrence of heat discomfort by 15%, with greater benefits observed in lightweight construction. Moreover, highly insulated buildings exhibited greater dependency on PCMs during the summer than poorly insulated ones [27]. Similar trends were noted in Curitiba (Cfb), where PCM usage notably reduced indoor maximum temperatures by 2.2 to 5.0 °C in lightweight construction, but had an insignificant impact on heavyweight constructions [16]. In this same climate, PCM application in lightweight housing models with high insulation consumed 3 to 5 times less energy than non-insulated scenarios [28], highlighting the significance of thermal insulation for energy efficiency in cold climates in Brazil.

While PCMs have demonstrated greater potential to mitigate energy consumption in buildings located in colder regions of Brazil, the absence of manufacturers and the need for adjustments in existing heavyweight systems pose challenges to their widespread adoption [16,29,30,31,32]. In contrast, lightweight construction systems have shown promise for effectively integrating PCMs, particularly when combined with Wood Frame technology, renowned for blending thermal and acoustic comfort with sustainability [33], which can be an effective approach to achieve passive conditioning in buildings [34].

Wood Frame construction, prevalent in Europe and North America, entails the use of processed reforested wood sheets, including plywood and Oriented Strand Board (OSB) panels, along with thermal insulation [33]. This approach holds promise in South America, particularly in Brazil, a leading wood product producer, enabling the adoption of this technology into Brazilian construction practices [35]. Moreover, Brazil’s abundant resources are rich in vegetable fat, including soybeans, castor beans, palm trees, and coconuts [36], with high saturated fatty acid content, which positions the country as a promising player in the development and utilization of bio-based PCMs for thermal storage in buildings [37].

Despite the growing attention toward PCMs in Brazil, a gap persists in national references regarding their use, especially in naturally ventilated buildings [38]. The need for research in tropical regions with hot and humid climates, notably in low latitudes like Brazil, is evident [39]. Hence, this study focuses on the specific combination of Wood Frame construction and PCM integration within the context of the Brazilian subtropical climate. This article presents a thermal performance analysis of naturally ventilated housing under the Temperate Oceanic Climate (Cfb) in a southern Brazilian city, where Wood Frame constructions and the growing popularity of PCMs are prominent. To this end, dynamic energy simulations were conducted for a typical Brazilian housing typology, both with and without PCM integration within the wooden frame envelope. Furthermore, the impact of adding a thermal insulation layer to the building’s external walls was examined. The results contribute to a better understanding of the thermal behavior of PCM-enhanced housing using Wood Frame strategies in the subtropical climate of Brazil.

## 2. Materials and Methods

### 2.1. Weather Conditions

**Figure 1:**
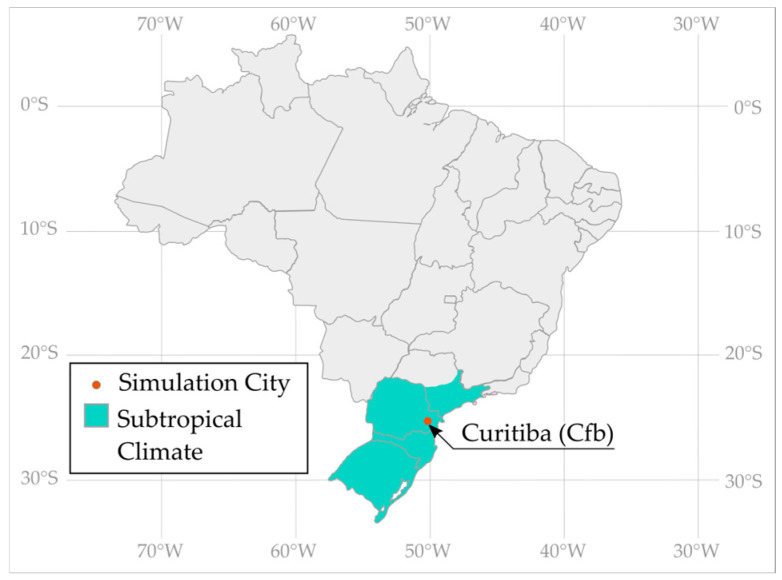
Geolocation of the selected city.

The subtropical climate of Brazil (Figure 1) was selected to evaluate PCM thermal performance due to its moderate temperatures, ideal for assessing PCM efficacy.

**Figure 2:**
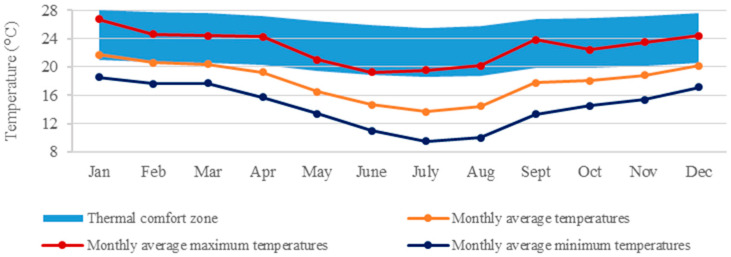
Monthly outdoor temperatures of Curitiba (Cfb).

Specifically, Curitiba-PR (25°25′42″ S, 49°16′24″ W), the nation’s coldest capital, was chosen for the case study (Table 1). With a Temperate Oceanic Climate (Cfb), as per Köppen’s classification, Curitiba experiences cold winters and significant heating demands (Figure 2) without a dry season [40]. However, heating systems are uncommon in Brazil. A 2019 report [41] revealed that only 0.37% of Brazilian households have heaters, with just 0.24% in the South.

### 2.2. Case Study Building

**Figure 3:**
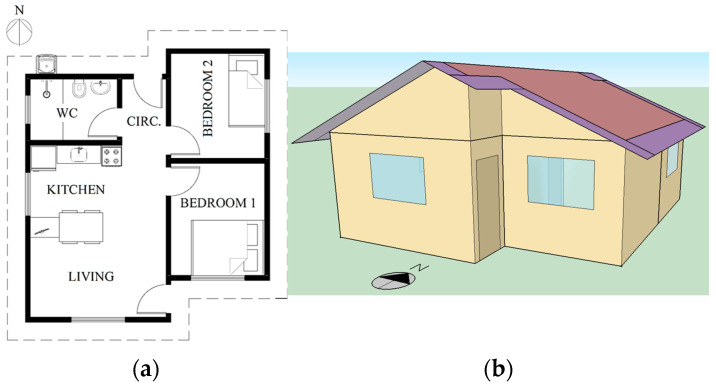
(**a**) Floor plan and (**b**) 3D image of the representative building design.

A 44.96 m^2^ detached house was chosen as a case study (Figure 3), representing the most common typology (35%) for single-family houses in Brazil, which make up 85% of the total dwelling stock [42]. It includes two bedrooms, a living room integrated into the kitchen, and a bathroom, as shown in Table 2 and Figure 3a. The glass area comprises 9.3% of the facade area, with no shading devices. The bedrooms, living room, and kitchen windows feature two horizontal sliding sashes with a ventilation factor (VF) of 0.45 and a lighting factor (LF) of 0.80. The bathroom window has framed glass louvres with a VF of 0.90 and LF of 0.65.

### 2.3. Envelope Characterization

In Brazil, the Standard NBR 15220-3 [43] outlines guidelines and construction strategies for passive thermal conditioning in summer and winter. It specifies appropriate design elements such as opening sizes, wall and roof types, and suitable materials. For Curitiba’s Cfb climate, it recommends lightweight external walls and lightweight insulated roofs [43]. Thus, a Wood Frame system consolidated in Brazil served as the basis for the case study, with thermal insulation only on the ceiling (Case A), following prior studies [44]. Additionally, a second model (Case B) was proposed to assess the effect of adding insulation to external walls on the building’s thermal behavior. Table 3 and Table 4 detail the building elements and thermal properties of each evaluated envelope model. Thermal Transmittance (U) and thermal capacity (CT) for each component were calculated according to NBR 15220-2 [45].

Glass thermal properties followed NBR 15575-1:2021 specifications, with a Thermal Transmittance (U) of 5.7 W/m^2^·K and a Solar Heat Gain Coefficient (SHGC) of 0.87 [46]. Window frame properties also adhered to NBR 15575-1:2021. All external and internal doors are wooden. As most typical Brazilian projects do not specify wall colors [42], reference values for solar absorptance (α) from NBR 15575-1 were used.

### 2.4. PCM Description

**Figure 4:**
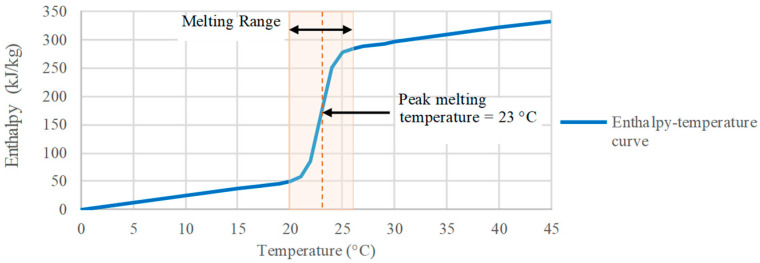
Enthalpy–temperature curve of analyzed PCM [48].

Previous studies have highlighted the potential of macroencapsulated bio-based PCM mats, applied to walls and ceilings, for enhancing thermal performance and energy efficiency in temperate climates [38]. Additionally, lower phase change temperatures were found to be more suitable for the Brazilian subtropical climate [47]. Analyzing the operative temperature results of the models without PCM, along with the average external temperature in Curitiba, provides a basis for devising effective PCM strategies. According to the recommendations of prior studies [17,18,19,20], the optimal melting point range for Curitiba’s climate (Cfb) lies between 20.4 °C and 23.4 °C. Therefore, an organic bio-based PCM, macroencapsulated in pouches made of white plastic sheeting, was selected for the case study, denoted as BioPCM. With a melting range from 20 °C to 26 °C, a peak melting point of 23 °C, and a latent heat of 223 kJ/kg (Figure 4), BioPCM was chosen based on its adequate thermo-physical properties for thermal storage in buildings, as provided by the manufacturer (Table 5). It was applied to the inner surface of external walls and/or above the drop ceiling prior to internal finishing layers.

### 2.5. Building Thermal Behavior

**Figure 5:**
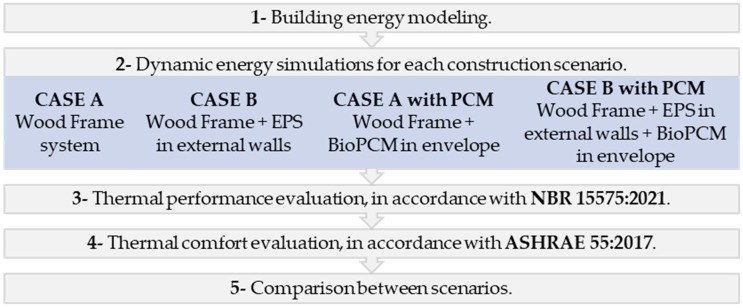
Flowchart of the methodological steps.

The evaluation of the dynamic thermal behavior of each construction scenario (Cases A and B with and without BioPCM) comprised two main analyses: assessing the dwelling’s thermal performance and evaluating the dwelling’s thermal comfort conditions for users during occupied hours. To establish a comparative basis, a prior study [47] examining the thermal behavior of a traditional Brazilian housing construction, typically comprising brick walls, was considered. Figure 5 delineates the process flowchart for the thermal behavior evaluation.

#### 2.5.1. Thermal Performance Evaluation

**Figure 6:**
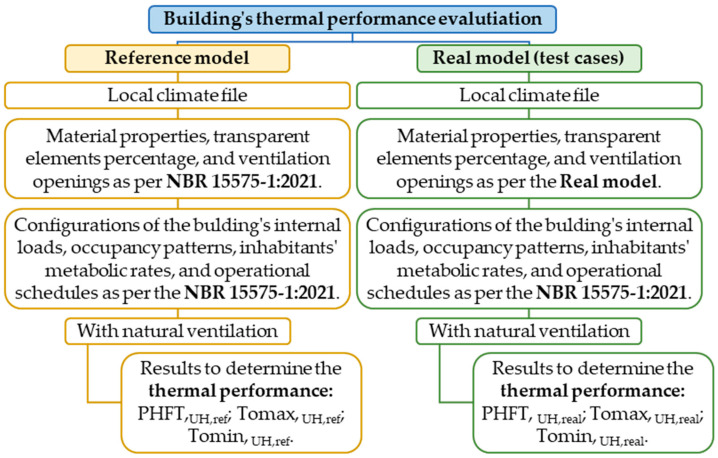
Procedures for evaluation of building’s thermal performance [46].

In the initial analysis, the dwelling’s thermal performance was assessed following the simulation method outlined in the Brazilian Standard NBR 15575-1:2021 for naturally ventilated environments, as depicted in Figure 6.

This method involves comparing the annual thermal performance of a Real Model building envelope (representing the building under evaluation) with that of a Reference Model building envelope established by the standard. Both models share identical hourly internal loads from occupancy, artificial lighting, and equipment usage in long-stay environments (APP) and have the same volumetric characteristics and contour conditions. However, the Reference Model requires adjustments to building material thermal properties and the proportions of transparent elements and openings based on reference values established by the standard [46].

Parameters such as the percentage of annual occupied hours in the acceptable temperature range (PHFT_,APP_), maximum annual operative temperature (Tomax_,APP_), and minimum annual operative temperature (Tomin_,APP_) were calculated for each long-stay environment (APP) in both the Real and Reference Models. These parameters were then used to derive corresponding parameters for the entire housing unit (UH) [46].

To meet the minimum (M) level of thermal performance, the Real Model must exhibit specific criteria in comparison to the Reference Model, as specified by Equations 1 to 3 in NBR 15575-1:2021 [46].

PHFT,_UH,real_ > 0.90 × PHFT,_UH,ref_ (1)

Tomax,_UH,real_ ≤ Tomax,_UH,ref_ + ∆Tomax (2)

Tomin,_UH,real_ ≥ Tomin,_UH,ref_ − ∆Tomin (3)

These criteria include (1) maintaining at least 90% of annual occupied hours within an acceptable temperature range (PHFT,UH,real) compared to the Reference Model (PHFT,UH,ref); (2) ensuring the maximum annual operative temperature (Tomax,UH,real) does not exceed that of the Reference Model by more than a maximum tolerance value (ΔTomáx), equal to 2 °C for single-family dwellings; and (3) guaranteeing the minimum annual operative temperature (Tomin,UH,real) is equal to or higher than that of the Reference Model after adjusting for a minimum tolerance value (ΔTomín), set at 1 °C for any evaluated UH [46].

#### 2.5.2. Thermal Comfort Evaluation

The impact of PCM usage in Cases A and B was assessed by comparing hourly indoor temperature data and evaluating occupants’ thermal comfort conditions during occupied hours. This analysis followed the adaptive comfort model outlined in ASHRAE 55 Standard [49], with 80% acceptability for neutral temperature. Subsequently, results were analyzed across scenarios to determine BioPCM’s effect on thermal comfort conditions.

It is important to highlight that the impacts of climatic variations, such as humidity and precipitation during the rainy season, on the thermal performance of PCMs in the surrounding environment have not been evaluated.

#### 2.5.3. Dynamic Energy Simulation

Building energy modeling for the Real and Reference Models utilized SketchUp Make 2016 (16.1.1449) software, OpenStudio plug-in (1.12.0), and Euclid extension (0.9.3). Dynamic energy simulations adhered to NBR 15575-1:2021 guidelines. Weather data of the Typical Meteorological Year (TMYx 2007-2021) for Curitiba [50] were used as input. All simulations provided hourly operative temperature data for each APP (To,APP) and hourly outdoor dry-bulb temperature data for each scenario over the course of one year.

A total of five dynamic simulations were conducted using EnergyPlus software version 8.9.0. These included four scenarios involving PCM usage (Cases A and B with and without BioPCM) and one corresponding to the Reference Model simulation. Thermal properties of building materials and the utilization of Phase Change Materials (PCMs) were adjusted using EnergyPlus software for each proposed building scenario.

The housing unit was assumed to accommodate four individuals, with a maximum of four people in the living/kitchen area and two in each bedroom. Occupancy, lighting, equipment usage, and other relevant factors followed specifications outlined in NBR 15575-1:2021 and recommendations from the ASHRAE 55 Standard (Table 6, Table 7 and Table 8).

In the simulations, all external walls were exposed to direct solar radiation, without shading on openings and no designed airflow rate into thermal zones, as the original project lacked shading devices or HVAC systems. Given the study’s focus on hypothetical building, certain simplifications were necessary, such as disregarding the influence of the surrounding environment and the degradation of external coatings in the simulations. The AirflowNetwork model in EnergyPlus simulated airflow between building zones using the Surface Average Calculation method to compute wind pressure coefficients on building surfaces. Natural ventilation protocols followed NBR 15575-1:2021 guidelines: window openings were restricted to occupancy periods in long-stay environments (APP), triggered by indoor dry-bulb temperatures exceeding 19 °C or surpassing outdoor dry-bulb temperatures; bathroom windows remained open year-round; internal doors were kept open, except for bathroom doors, which stayed closed; and external doors also remained closed throughout the year [46]. Moreover, the AirflowNetwork:MultiZone:Component:DetailedOpening object in EnergyPlus defined airflow properties through windows and doors. This involved using coefficients and exponents governing air mass flow, as per NBR 15575-1:2021, to regulate air leakage through window and door frames [46]. Also, ventilation factors (VF) derived from the building under study determined window width and height opening factors.

Heat transfer between floors and ground was simulated using the Site:GroundDomain:Slab object in EnergyPlus, employing undisturbed ground temperatures based on the Finite Difference model to solve for the ground temperatures [51]. A fixed solar orientation at 0° azimuth was adopted, representing the worst thermal performance scenario for the selected housing model [42], to explore the PCM’s potential in enhancing thermal performance under challenging conditions.

To allow dynamic simulations with PCM in EnergyPlus, the Conduction Finite Difference (CondFD) algorithm was employed. This algorithm discretizes construction elements at various nodal points (Δ*x*) and solves heat transfer equations numerically for each node. The fully implicit scheme was chosen in the CondFD for the heat transfer formulation procedures, as shown in Equation (4) [51].

Next, node enthalpies are updated in each new time iteration and then used to develop an equivalent Cp variable, considering the phase change process, by incorporating Equation (5) [51]. For that, the enthalpy–temperature (h-T) curve of the BioPCM was introduced as an input into the CondFD model to account for the material’s specific heat as a function of temperature. Additionally, default values of 3 for the Space Discretization Constant and a time step (Δ*x*) of 3 min were selected to ensure acceptable annual results for the simulations [52]:

(4) $$C_{p}\rho ∆x\frac{T_{i}^{j+1}-T_{i}^{j}}{∆t}=\left(k_{W}\frac{T_{i+1}^{j+1}-T_{i}^{j+1}}{∆x}+k_{E}\frac{T_{i-1}^{j+1}-T_{i}^{j+1}}{∆x}\right)$$

(5) $$C_{p}=\frac{h_{i,new}-h_{i,old}}{T_{i,new}-T_{i,old}}$$

where $C_{p}$ = specific heat of material; ρ = density of material; ∆x = Finite Difference layer thickness; ∆t = calculation time step; T = node temperature; h = node enthalpy; _*i*_ = node being modeled, while nodes _*i*+1_ and _*i*−1_ denote the adjacent nodes on the interior and exterior sides of the construction, respectively; ^*j*^ and ^*j*+1^ represent the previous and new time steps, respectively; $k_{W}$ = thermal conductivity for interface between _*i*_ node and _*i*+1_ node; and $k_{E}$ = thermal conductivity for interface between _*i*_ node and _*i*−1_ node [51].

Validation is a critical step in providing reliable results for heat transfer and thermal comfort in simulated buildings. The CondFD algorithm in EnergyPlus is among the most widely used and validated models for assessing the thermal behavior of PCMs [53]. Extensive validation studies [12,27,54,55,56,57,58,59] of EnergyPlus’s PCM module have demonstrated high consistency between numerical simulations and experimental data, reinforcing its credibility for such analyses.

## 3. Results

### 3.1. Impact of PCM on Indoor Temperatures

**Figure 7:**
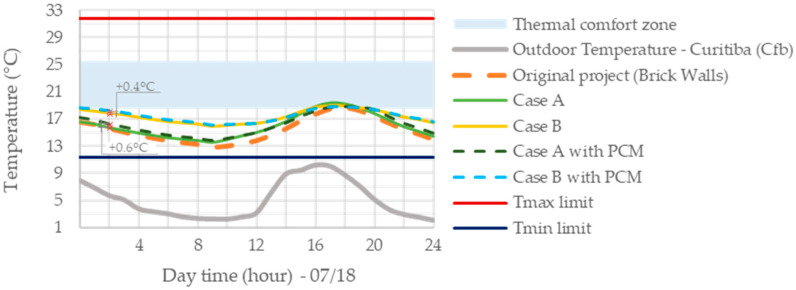
Thermal behavior of Bedroom 2 on the coldest day of the year across evaluated scenarios.

Figure 7 illustrates the indoor temperatures in Bedroom 2 (north-oriented) on 18 July, the coldest day of the year for Curitiba-PR’s climate (Cfb), obtained from simulations. Bedroom 2 exhibited the poorest thermal behavior in the house across all evaluated scenarios. The solid red and dark blue lines represent the temperature limits (Tmax and Tmin), determined through the simulation method outlined in NBR 15575-1. The light blue area indicates the acceptable thermal comfort temperature range for users, derived from the adaptive comfort model of the ASHRAE 55 Standard. The results for Wood Frame construction systems without BioPCM (Case A) and with BioPCM (Case A with PCM) are depicted in solid and dashed green lines, respectively. Moreover, the results for Wood Frame construction with EPS insulation in external walls are shown in yellow for the scenario without BioPCM (Case B) and in light blue for the scenario with BioPCM (Case B with PCM). Furthermore, the dashed orange line corresponds to thermal data from previous studies [47] conducted on the original building project (brick walls).

Although all scenarios show thermal discomfort conditions due to cold during occupied hours (Figure 7), those with thermal insulation in the envelope (“Case B” and “Case B with PCM”) exhibited better thermal performance by reducing heat losses from indoor to outdoor environments. However, integrating BioPCM into the envelope did not significantly raise indoor temperatures on the coldest day, with increases up to 0.6 °C compared to “Case A” and up to 0.4 °C compared to “Case B”. This is because external temperatures remained below BioPCM’s melting point, preventing its melting during sunny hours and the subsequent release of stored heat through its solidification [14]. Consequently, BioPCM’s potential benefits were not fully realized in these conditions.

**Figure 8:**
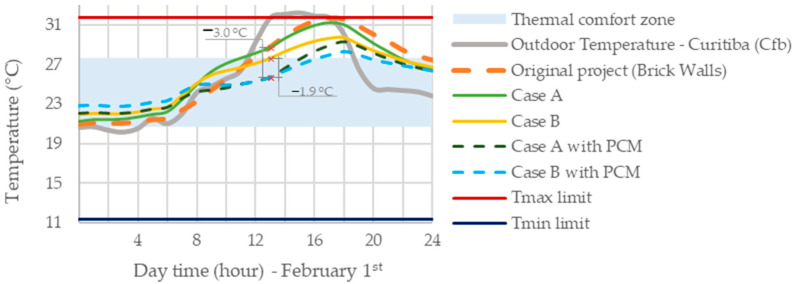
Thermal behavior of Bedroom 2 on the hottest day of the year across evaluated scenarios.

Similarly, Figure 8 illustrates the indoor temperatures in Bedroom 2 on 1 February, the hottest day within the simulated typical year for Curitiba’s climate (Cfb). The brick wall model demonstrated the poorest thermal performance, corroborating the need for changes in the envelope of the building’s original design. Nonetheless, the Wood Frame system (Case A) exhibited the least impact on reducing maximum indoor temperatures compared to the other Wood Frame construction scenarios. However, integrating BioPCM reduced indoor temperatures on the hottest day by up to 3.0 °C compared to “Case A” and up to 1.9 °C compared to “Case B”. Comparing scenarios without BioPCM (Case A and Case B), the inclusion of EPS in Wood Frame walls led to a greater potential in reducing maximum indoor temperatures during Curitiba’s summer (Cfb). Similarly, when comparing scenarios “Case A with PCM” and “Case B with PCM”, adding EPS in external walls improved the effectiveness of BioPCM in reducing thermal amplitude within the building. Conversely, when contrasting scenarios “Case B” and “Case B with PCM”, integrating BioPCM into the building envelope enhanced the thermal insulation performance during summer. These findings are consistent with those reported by other authors [10,27,28].

From Table 9, it can be observed that the simple exchange of the original building system (brick walls) to the Wood Frame construction (Case A) presented the slightest effect on the indoor temperature ranges throughout the year, providing a reduction of up to 0.7 °C at maximum temperatures and an increase of up to 1.4 °C at minimum temperatures in the house. However, this strategy provided the least impact by reducing the daily thermal amplitude by up to 1.2 °C when compared to the other scenarios. Indeed, it still showed a higher occurrence of cases with temperatures above the acceptable occupants’ thermal comfort zone, as highlighted in the light blue area in Figure 8. Meanwhile, the inclusion of thermal insulation (EPS) in the external walls in the PCM-enhanced building (Case B with PCM) demonstrated the greatest building thermal performance, providing a reduction of up to 2.6 °C and an increase of up to 4.1 °C at the maximum and minimum temperatures, respectively.

Incorporating BioPCM into the envelope of the Wood Frame construction system without thermal insulation in the walls (Case A with PCM) led to a maximum reduction of up to 4.25 °C in the daily thermal amplitude compared to the scenario without the use of these materials (Case A). Meanwhile, in the model with a higher level of thermal insulation (Case B with PCM), this reduction was up to 3.9 °C compared to the model without the use of BioPCM (Case B).

Comparing the scenarios without PCM, from Case A to Case B, the EPS inclusion in the Wood Frame walls leads to a greater potential in increasing the minimum temperatures and reducing the indoor thermal amplitude. Conversely, BioPCM addition to the Wood Frame system proved more effective in reducing daily thermal amplitude in all scenarios, surpassing the impact of EPS. Indeed, the combination of BioPCM with EPS demonstrated the greatest potential for reducing indoor thermal amplitude, up to 6.4 °C compared to the original scenario, enhancing the thermal insulation performance [10].

### 3.2. Thermal Behavior Analysis

**Figure 9:**
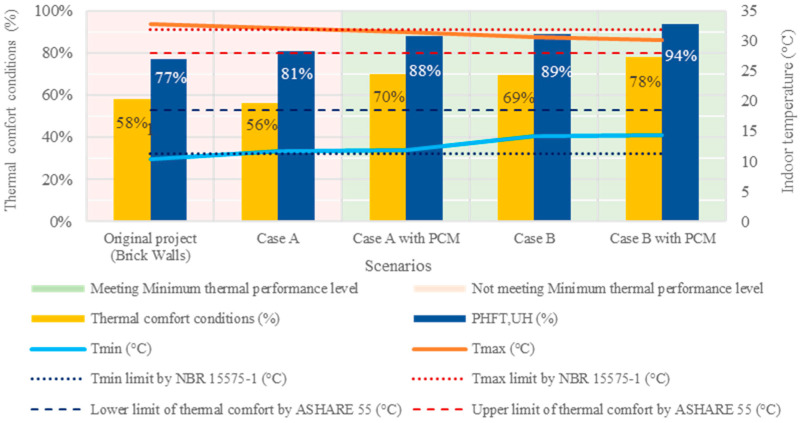
Summary of building’s thermal performance and thermal comfort condition evaluation during occupied hours throughout the year in Curitiba (Cfb).

Figure 9 summarizes the building’s thermal behavior throughout the year in Curitiba (Cfb) for all assessed scenarios. Their compliance with the Brazilian Thermal Performance Standard NBR 15575-1 is denoted by green and red highlights for meeting or not meeting the standard, respectively. Additionally, the figure illustrates the average percentage of hours within thermal comfort conditions during occupied hours for each evaluated scenario.

Consistent with prior studies [47], the original building, in its initial state, fell short of meeting the minimum thermal performance level for Curitiba’s climate, failing to satisfy any of the mandatory criteria outlined in NBR 15575-1. Transitioning to the Wood Frame system in “Case A” (Figure 9) also proved to be an insufficient strategy to achieve the minimum level of thermal performance, notably due to the failure to meet the Tmax requirement. In fact, this strategy slightly worsened thermal comfort conditions, with a 1.7% decrease compared to the original building (brick walls), despite a 3.5% improvement in the criteria of percentage of annual occupied hours within the acceptable temperature range (PHFT_,UH_). Similar trends were observed when comparing scenarios from “Case A with PCM” to “Case B”, highlighting discrepancies between NBR 15575-1 and ASHRAE 55 criteria. These discrepancies are attributed to variations in temperature thresholds between the two standards, as evidenced in Figure 9 and noted in previous studies [47], with ASHRAE 55 exhibiting higher and less permissive thresholds compared to NBR 15575-1.

However, all other evaluated scenarios met the Brazilian standard, underscoring the positive influence of BioPCM and/or EPS applications in Wood Frame systems. The addition of thermal insulation to external walls (Case B) notably improved thermal comfort conditions by up to 11.3%, despite not being among the passive thermal conditioning strategies recommended by NBR 15220-3 for Curitiba’s subtropical climate (Cfb).

In all cases, the integration of BioPCM into the building envelope improved thermal performance and occupants’ thermal comfort conditions under Curitiba’s climate (Cfb). Indeed, the inclusion of BioPCM yielded superior results compared to EPS alone in cases A and B, as detailed in Table 10. In “Case A with PCM”, BioPCM increased the percentage of occupied hours within thermal comfort conditions by 13.8% compared to “Case A” without PCM, while the inclusion of EPS alone in external walls improved thermal comfort conditions by 13.0% in “Case A”. These findings align with the literature emphasizing the efficacy of PCM in low-thermal-inertia buildings [16,27]. “Case B with PCM” exhibited even better results, with a 20.0% increase compared to the original building (brick walls) and an 8.8% increase compared to “Case B” without PCM, supporting previous findings on the beneficial impact of PCM on the thermal performance of building insulation [10,28].

**Figure 10:**
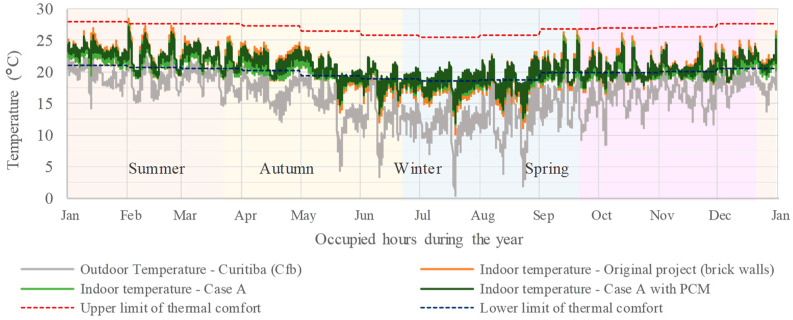
Bedroom 2 temperatures throughout the year during occupied hours for Case A, Case A with PCM, and the original scenario.

**Figure 11:**
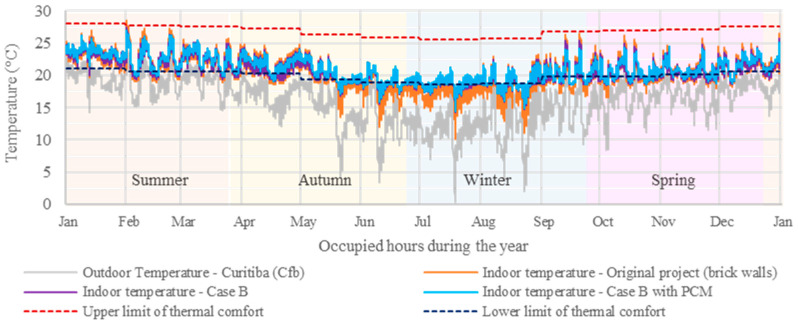
Bedroom 2 temperatures throughout the year during occupied hours for Case B, Case B with PCM, and the original scenario.

Figure 10 and Figure 11 present the annual indoor temperatures for Bedroom 2 in Curitiba’s climate (Cfb) during occupied hours, comparing the effects of BioPCM in “Case A” and “Case B” scenarios to the original brick wall construction. In both situations, BioPCM’s effectiveness varied by season, showing greater benefits in summer and autumn when outdoor temperatures exceeded its melting range, thus reducing heat discomfort and increasing minimum indoor temperatures. However, its performance declined in winter and spring, particularly in “Case A” (Figure 10), where it did not contribute significantly to increasing minimum indoor temperatures due to frequent low outdoor temperatures and significant daily thermal variations, aligning with previous studies [12,26,47,60]. Conversely, “Case B with PCM” (Figure 11) demonstrated improved thermal comfort in cold conditions, likely due to the thermal insulation contribution [27,28], proving to be the most effective strategy among the evaluated scenarios in Curitiba’s climate, where heating demands are more pronounced. It is worth noting that these simulations did not account for systems that could enhance PCM efficiency, such as night ventilation or ambient temperature control, as their implementation is uncommon in Brazilian housings.

**Figure 12:**
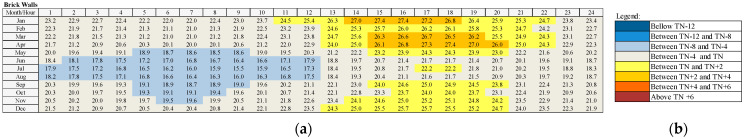
(**a**) Hourly monthly indoor temperature data for Bedroom 2 throughout the year under the original project scenario (brick walls); (**b**) graph legend.

**Figure 13:**
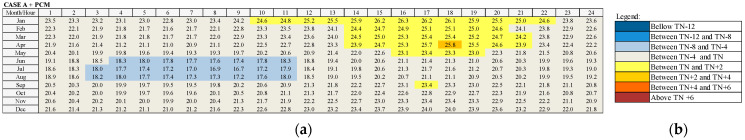
(**a**) Hourly monthly indoor temperature data for Bedroom 2 throughout the year under Case A with PCM scenario; (**b**) graph legend.

**Figure 14:**
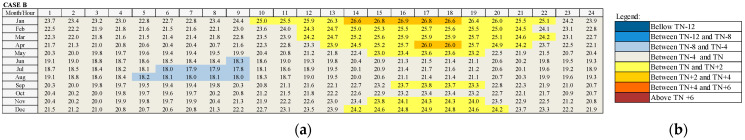
(**a**) Hourly monthly indoor temperature data for Bedroom 2 throughout the year under Case B scenario; (**b**) graph legend.

**Figure 15:**
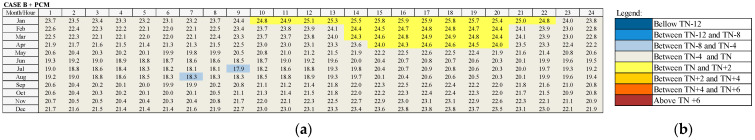
(**a**) Hourly monthly indoor temperature data for Bedroom 2 throughout the year under Case B with PCM scenario; (**b**) graph legend.

Figure 12, Figure 13, Figure 14 and Figure 15 present the average indoor temperature by hour throughout the year for Bedroom 2 of the building in its original design, Case A with PCM, Case B, and Case B with PCM. The color scale in these figures indicates temperature intervals relative to the neutral temperature (TN), as defined by ASHRAE 55:2017.

Case A+PCM (Figure 13) demonstrated an improvement in thermal comfort during heat conditions compared to the original building system (Figure 12), particularly between 1:00 p.m. and 8:00 p.m. during the warmer months, which include January to April and September to December. Additionally, the system improved thermal discomfort conditions for cold between 5:00 a.m. and 9:00 a.m. from May to November and during the early morning hours from June to August.

On the other hand, Case B+PCM (Figure 15) demonstrated an improvement in high indoor temperatures between 2:00 p.m. and 9:00 p.m. during the transition months of April and May. Furthermore, there was a significant reduction in cold indoor temperatures between 3:00 a.m. and 11:00 a.m. during the colder months, particularly from June to August.

When comparing the hourly monthly temperature data between the scenarios without PCM (Case B), in Figure 14, and with PCM (Case B+PCM), in Figure 15, it is evident that the addition of PCM to the construction system with EPS effectively improved thermal discomfort conditions for cold between 5:00 a.m. and 9:00 a.m. during the colder months from June to August.

**Figure 16:**
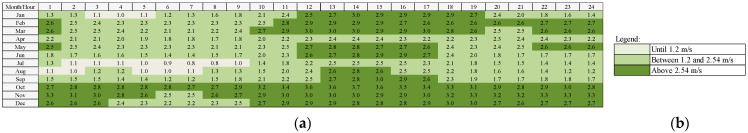
(**a**) Hourly monthly wind speed data throughout the year; (**b**) graph legend.

**Figure 17:**
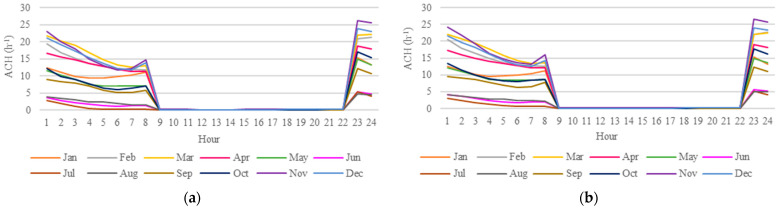
Hourly monthly ACH distribution data for Bedroom 2 throughout the year under (**a**) Case A with PCM scenario and (**b**) Case B with PCM scenario.

Since indoor temperatures in buildings are strongly influenced by airflow, an hourly distribution analysis by month of Air Changes per Hour (ACH) and wind speed was carried out to assess the current impact of PCM on heat gains in Bedroom 2. Bedroom 2 window operation was restricted to occupancy periods (12:00 a.m.–7:59 a.m. and 10:00 p.m.–11:59 p.m.) and was triggered by indoor dry-bulb temperatures exceeding 19 °C or surpassing the outdoor dry-bulb temperatures. Figure 16 illustrates higher wind speeds during periods when indoor temperatures were lower. Conversely, Figure 17 reveals no variation in the ACH distribution between 9:00 a.m. and 10:00 p.m., as Bedroom 2 remained sealed during those hours due to non-occupancy. These findings suggest that PCM contributed significantly to reducing heat gains in the room during the hottest months by increasing the envelope thermal resistance and absorbing thermal energy that would otherwise be transmitted to the room due to its melting temperature range.

On the other hand, Figure 17 shows lower ACH distributions during the colder months, from June to August, which may have contributed to reduced heat losses in Bedroom 2. However, no significant differences were observed between the ACH distributions in the scenarios without EPS (Case A+PCM) and with EPS (Case B+PCM). Thus, the improvement in thermal comfort conditions for cold between the two scenarios could be attributed to the addition of EPS and/or PCM to the building system, as there was no significant alteration in air distribution within the room during this period.

In conclusion, adding PCM to the building system significantly enhanced thermal comfort in Bedroom 2, particularly during the hottest and coldest periods of the year. The analysis indicates that PCM effectively reduced heat gains during the warmer months and minimized heat losses during colder periods, even without notable changes in air infiltration rates. These findings highlight PCM’s role in improving indoor thermal conditions, with additional benefits observed when combined with EPS.

## 4. Discussion

This study illustrates that typical Brazilian dwellings, consisting of brick walls, display inadequate thermal performance within Curitiba’s climatic conditions (Cfb), necessitating significant heating requirements for much of the year. Transitioning from masonry walls to the Wood Frame construction alone, without BioPCM or EPS, failed to meet the minimum thermal performance level mandated by NBR 15575-1. Noteworthy disparities in practice emerged between the criteria stipulated in the NBR 15575-1 and ASHRAE 55 Standards. While certain scenarios exhibited improvements in thermal performance, they concurrently worsened the percentage of occupied hours within thermal comfort conditions for users. Introducing EPS to external walls effectively raised minimum temperatures, whereas incorporating BioPCM notably reduced daily thermal amplitude in Wood Frame construction, thereby enhancing thermal comfort conditions.

The most promising results in enhancing building thermal behavior were achieved by combining BioPCM with thermal insulation (EPS) on the inner surface of the building envelope. Nonetheless, thermal discomfort conditions persisted primarily during colder seasons, indicating the insufficiency of this strategy to consistently improve building thermal performance throughout the entire year. This outcome was expected, given the substantial and abrupt daily outdoor temperature fluctuations characteristic of Curitiba (Cfb) during these periods, jeopardizing the completion of BioPCM’s charging and discharging cycles [31]. In addition, the elevated relative humidity levels (approximately 88%), coupled with reduced solar radiation during Curitiba’s winter, may restrict the efficacy of PCM in improving building thermal behavior by reducing or delaying heat transfer between the environment and PCM [12,13,14]. Moreover, indoor temperatures during cold seasons often fell below the melting range of BioPCM (20 to 26 °C), rendering BioPCM nearly inactive during these periods, as they were not completely melted enough [12]. Consequently, it is advisable to enhance BioPCM’s effectiveness by integrating additional passive solar heating strategies, such as employing internal walls with greater thermal inertia or exposing floors to direct solar radiation, as proposed by Brazilian building standards [43]. Furthermore, exploring alternative PCMs with higher latent heat storage capacities or varying melting points tailored to different seasons is recommended [16,26].

It is preferable to utilize eutectic compositions with sharper melting points rather than those with broader temperature ranges, such as the PCM applied in this study. This allows for applications requiring constant indoor temperature regulation, facilitating the precise control of thermal energy storage and release. Such materials typically exhibit enhanced phase stability and reduced phase separation, improving their long-term performance and reliability in building applications [61,62]. In a parametric optimization study on BioPCM, authors [47] demonstrated that optimal thermal comfort conditions for Curitiba’s (Cfb) climate are achieved by using PCMs with lower melting temperatures (23 °C) and higher latent heat–area values (1250 kJ/m^2^) applied to the inner surfaces of the entire building envelope (roof and walls), especially in lightweight and insulated construction models. Notably, thermal conductivity was the only PCM property whose variation showed no significant impact on thermal comfort results. However, increasing thermal conductivity proved to be an effective strategy for compensating for the lower thermal performance of PCMs with reduced latent heat–area values.

These findings provide critical physical parameters that material scientists can use to synthesize and develop advanced PCMs tailored for enhanced thermal performance in building applications.

## 5. Conclusions

This study evaluated the efficacy of Phase Change Materials (PCMs) in enhancing the thermal performance of naturally ventilated houses and their ability to ensure occupants’ thermal comfort under the Brazilian subtropical climate. Through a case study of a typical single-family housing model in Curitiba’s climatic conditions (Cfb), this study addressed two primary research questions: (1) the effectiveness of the Wood Frame construction system in improving building thermal performance and percentage of hours within thermal comfort conditions during occupied hours for users; and (2) the impact of applying PCM and/or thermal insulation into the building envelope on the thermal behavior of houses under the Brazilian subtropical climate.

Employing computational simulation methodologies and adhering to the criteria outlined in NBR 15575-1 and ASHRAE 55, four hypothetical scenarios of Wood Frame system utilization were assessed and compared for Curitiba-PR’s climate (Cfb), considering (i) the presence or absence of EPS on external walls (“Case A” and “Case B”) and (ii) the inclusion or exclusion of BioPCM with a melting point of 23 °C within the building envelope (“Case A with PCM” and “Case B with PCM”). Additionally, previous studies on the thermal performance of the building in its original design, comprising ceramic masonry walls, were incorporated for comparative analysis, revealing a failure to meet the minimum criteria stipulated by Brazilian standards.

The findings indicate that the Wood Frame system without thermal insulation in the external walls, and without BioPCM in the building envelope, did not prove to be advantageous for maintaining internal temperatures of houses within the recommended thermal comfort range for the climate throughout the year. Conversely, the evaluated BioPCM emerged as an effective strategy for this purpose, particularly when combined with thermal insulation, capable of enhancing thermal comfort conditions by up to 20.0% and reducing the daily thermal amplitude by 6.4 °C compared to the original building (brick walls). However, the impact of BioPCM varies with seasonality, predominantly in winter, where the BioPCM-enhanced building still experiences thermal discomfort due to cold.

The results underscore the importance of integrating passive solar heating strategies to enhance the effectiveness of PCM heat storage during colder seasons in the subtropical Brazilian climate. This study makes an academic contribution by exploring PCM applicability in residential buildings within this climatic context, and a practical contribution by proposing an innovative approach to improve thermal performance.

Future research should examine the potential impacts of climate change on building performance, considering PCMs in future simulation scenarios. Cost–benefit analyses are also critical, addressing both the high acquisition costs of PCMs and the energy savings they generate, especially given their current lack of domestic production in Brazil. Furthermore, such analyses should consider the improved performance of PCM systems when paired with artificial ventilation or temperature control strategies.

Brazil’s rich biodiversity provides a unique opportunity to develop bio-based PCMs, positioning the country as a potential leader in this technology. However, economic feasibility studies remain essential for their widespread adoption. Raising market awareness about the benefits of PCMs and fostering their integration into the construction industry are key steps toward advancing sustainable building practices in Brazil.

## Figures and Tables

**Table 1:** Selected city for the case study [40].

City	Elevation	Average Annual Outdoor Temperature	Average Annual Relative Humidity	Annual Precipitation	Average Wind Intensity
Curitiba-PR	925 m	18.0 °C	81%	1575.8 mm	2.2 m/s

**Table 2:** Values for housing unit geometry.

Description	Dimensions
Floor-to-ceiling height	2.50 m
Living room	17.81 m^2^
Bedroom 1 (B1)	8.06 m^2^
Bedroom 2 (B2)	7.54 m^2^
WC	4.08 m^2^
Corridor	2.23 m^2^
Net area	39.73 m^2^

**Table 3:** Envelope building materials.

Description	External Walls		Roof
	Case A	Case B	Cases A and B
Construction Element	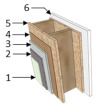	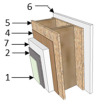	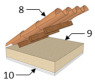
Thermal Transmittance (W/m^2^ K)	U = 1.93	U = 0.89	U = 0.37
Thermal Capacity (kJ/m^2^ K)	CT = 67.4	CT = 54.7	CT = 25.0
Solar Absorptance	α = 0.58	α = 0.58	α = 0.65

Legend: 1 = Acrylic finish; 2 = Base coat; 3 = Cement board; 4 = Pine wood; 5 = OSB; 6 = Gypsum board (wall); 7 = EPS; 8 = Roof tile; 9 = Glass wool; 10 = Gypsum board (roof).

**Table 4:** Thermal properties of envelope elements.

Element		Equivalent Thickness (cm)	Thermal Conductivity (W/m K)	Specific Heat (kJ/kg K)	Density (kg/m^3^)
1	Acrylic finish	0.30	1.15	1.00	2000
2	Base coat	0.50	1.15	1.00	1840
3	Cement board	0.80	0.35	1.00	1700
4	Pine wood	0.15	0.15	1.34	494
5	OSB	0.95	0.17	2.30	681
6	Gypsum board (wall)	1.25	0.35	0.84	750
7	EPS	2.50	0.04	1.42	25
8	Roof tile	0.60	0.65	0.84	1700
9	Glass wool	10.00	0.045	0.70	10.35
10	Gypsum board (roof)	2.50	0.35	0.84	750

**Table 5:** PCM thermo-physical properties [48].

	BioPCM
Product name	M-51 BioPCM^TM^
Peak nominal melting temperature (°C)	23
Latent heat capacity (kJ/kg)	~230
Specific heat capacity (J/kg K)	1970
Thermal conductivity (W/m K)	Liquid: ~0.15 Solid: ~0.25
Density (kg/m^3^)	860
Average thickness of the pouch (m)	0.0064
Latent heat–area (kJ/m^2^)	~580
Enclosure/pouch material	Plastic foil/white plastic sheeting
Pouch material thickness between the pouches (mm)	0.508

**Table 6:** Occupancy schedule and internal gains for occupants [46].

Zone	Occupation Period	Activity	Het Gains from People [W]	Fraction Radiant
Bedrooms	00 h 00 a.m.–07 h 59 a.m. 10 h 00 p.m.–11 h 59 p.m.	Sleeping or resting	81	0.30
Living	0 2h 00 p.m.–09 h 59 p.m.	Sitting or watching TV	108	0.30

**Table 7:** Usage schedule and internal gains for lighting systems [46].

Zone	Usage period	Installed Power Density [W/m^2^]	Fraction Visible	Fraction Radiant
Bedrooms	06 h 00 a.m.–07 h 59 a.m. 10 h 00 p.m.–11 h 59 p.m.	5.00	0.23	0.30
Living	04 h 00 p.m.–09 h 59 p.m.	5.00	0.23	0.30

**Table 8:** Usage schedule and internal gains for electric equipment [46].

Zone	Usage Period	Electric Equipment Design Level [W]	Fraction Radiant
Living	02 h 00 p.m.–09 h 59 p.m.	120	0.30

**Table 9:** Thermal behavior impacts for each evaluated scenario compared to the original building.

Maximum Impact	Case			
	A	A with PCM	B	B with PCM
Tmin increase (°C)	1.4	1.6	3.9	4.1
Tmax reduction (°C)	0.7	1.4	2.1	2.6
Daily thermal amplitude decrease (°C)	1.2	4.5	3.9	6.4

**Table 10:** Comparison of thermal comfort conditions during occupied hours for users in Wood Frame scenarios.

Comparison Between Cases	Thermal Comfort Condition Impact (%)	
B–A	EPS effect	+13.0%
(B with PCM)–(A with PCM)		+8.0%
(A with PCM)–A	BioPCM effect	+13.8%
(B with PCM)–B		+8.8%

## Data Availability

Data are contained within the article.
